# Prison inmates with court-ordered treatments: are they really different?

**DOI:** 10.1186/s12991-022-00382-6

**Published:** 2022-02-11

**Authors:** Isabella D’Orta, François R. Herrmann, Panteleimon Giannakopoulos

**Affiliations:** 1 Division of Institutional Measures, Medical Direction, Geneva University Hospitals, Geneva, Switzerland; 2 Department of Rehabilitation and Geriatrics, Geneva University Hospitals and University of Geneva, Geneva, Switzerland; 3 Department of Psychiatry, University of Geneva, Geneva, Switzerland

**Keywords:** Court-ordered treatments, Criminal status, Prison, Psychiatry

## Abstract

**Background:**

Both the frequency of court-ordered treatments (COT) for offenders and prevalence of mental disorders among regular prison inmates steadily increased in most western countries. Whether there are major sociodemographic and clinical differences between these two populations is still matter of debate.

**Methods:**

We compared the sociodemographic and clinical characteristics in a representative sample of 139 regular prison inmates versus 61 offenders with COT admitted during a 5-year period in an acute psychiatric care unit located in the central prison of the Geneva county. Fisher exact, unpaired Student’s *t* and Mann–Whitney *U* tests were used to compare demographic and clinical variables between COT patients and regular inmates. In addition, univariate and multivariable ordered logistic regression models were built to identify the sociodemographic and clinical determinants of COT.

**Results:**

COT patients were significantly older, less frequently married, with better education attainment, predominantly French-speaking, of the Christian religious group and with regular religious practice. History of psychiatric outpatient care was significantly more frequent in this group. Unlike the significantly higher occurrence of adjustment disorders in regular prisoners, psychosis was the main diagnosis in COT patients. When all diagnostic categories were taken into account in multivariable models, the presence of personality and psychotic disorders were the stronger predictors of COT status.

**Conclusions:**

Our data reveal that offenders with COT represent a clinically distinct group with an overrepresentation of personality and psychotic disorders. Moreover, they show that, at least in the Swiss penitentiary system, COT patients are less exposed to acculturation issues compared to regular inmates.

## Introduction

High levels of psychiatric morbidity are well documented in prisons and are frequently associated with violence, victimization and self-harm [1]. There is evidence that, in some countries the prevalence of mental disorders among prison inmates is even higher than in psychiatric facilities, yet they remain poorly diagnosed and treated [2]. Although the principles of treatment of mentally disturbed offenders vary substantially among European countries, two main tracks can be identified. The first concerns criminally responsible offenders that receive the requisite psychiatric treatment via psychiatric consultations on a voluntary basis [3–5]. When needed, inpatient mental health care for this population may be provided via voluntary or compulsory admission to psychiatric hospitals [6, 7] or, more rarely, in prison psychiatric wards [8]. The second refers to offenders with decreased responsibility or high risk of recidivism due to long-lasting mental disorders identified via psychiatric expert witness. This population may be compulsory admitted to psychiatry care instead of an ordinary sequence under court-ordered treatments (COT) that take place in outpatient facilities or in high and medium-security hospitals [8–10]. These hospitals may be or not located in prison, are usually, but not consistently, funded by the health system and did not depend on the penitentiary system. Two distinct COT procedures are illustrated by the French and German examples. In France, COT were introduced under the form of injunction to care in order to lower the risk for recidivism on the premise that many offenders are affected by psychiatric disorders. It mainly concerns sex offenders with paraphilia but also other psychiatric pathologies such as mental retardation and personality disorders treated in outpatient settings [5]. In Germany, COT also encompasses inpatient care in high and medium-security hospitals in the federal states that provide a therapeutic environment suited for the specific needs of forensic psychiatric patients in long-term settings [3]. The investment in such inpatient settings for COT (as opposed to the usual outpatient care of regular inmates) is also found in Switzerland. A single medium-security hospital in Geneva central prison, referred to as Curabilis, is in charge of intensive COT inpatient treatments for inmates from all French and Italian-speaking Swiss counties. The admission in Curabilis needs a psychiatric expertise conducted during the prosecution of a serious crime. Care programs include psychotropic medication, individual and group psychotherapy, work therapy, and psychomotricity. Medial and nurse teams work in close collaboration with detention officers but they are independent on the penitentiary system. Curabilis also includes a unique psychiatric ward for acute treatment of both regular inmates and COT patients for the same catchment area (UHPP; Unité hospitalière de psychiatrie pénitentiaire).

Whether or not COT patients differ from patients admitted in psychiatric hospitals and regular inmates with acute mental disorders is a matter of intense debate [11]. For some authors, the legal framework implies an artificial distinction between COT patients and the other two populations [12, 13]. However, previous analyses showed significant differences in the demographic and clinical profile of COT patients in high and medium-security hospitals compared to patients treated in general psychiatry. They are more often single, with higher suicide risk [14], more frequent psychotic beliefs [15], lower education attainment and occupational levels [16, 17].

The main objective of our study is to explore the differences between COT and regular inmates taking the advantage of their coexistence in the UHPP. In particular, we aimed to examine whether these two populations differ in terms of clinical profile, social support, religion beliefs and previous psychiatric history when they are admitted for intensive care during their imprisonment. Our a priori hypothesis is that COT patients display severe and long-term evolving psychiatric disorders, and are less exposed to the adverse effects of immigration and acculturation compared to regular inmates. We had the opportunity to compare the demographic, social and clinical characteristics of COT patients and regular inmates in a representative sample of offenders who were admitted in this unit during a five-year period (2014–2019).

## Materials and methods

### Study sample and data collection

We examined the psychiatric records corresponding to all admissions in UHPP during a 5-year period (2014–2019). The total mean number of admissions per year for the period of reference was of 261. Importantly, there is no crisis discharge in this unit since psychiatric admissions cannot be refuted and number of beds is usually sufficient to cover the needs of acute care. In the rare cases of bed lacking, the hospital stays take place in a general psychiatry unit. Patients are admitted to the UHPP in the presence of acute symptoms associated with self or others-threatening behavior and need for urgent psychiatric care. In order to avoid the confounding effect of treatment discontinuation and active drug addiction, these cases were excluded from the present analysis. Multiple admissions were registered during the reference period in 71.3% of cases (*n* = 930). To prevent overweighting of those repeatedly admitted, we randomly selected a hundred cases for each group (repeated and single admissions). The final sample included 200 cases (mean age: 32.8 ± 10.3, age range: 20–44). Among them, there were 139 regular prison inmates versus 61 offenders with COT.

Each patient was assigned an identification number that was derived from the name and birth date and subsequently encrypted. Sociodemographic data included age, gender, marital status (at initial admission), education attainment (binary variable based on obligatory versus high school education; cut-off: 9 years), most frequently speaking language (French, English or Arabic), religious group (none, Christian, Muslim, other), regular religious practice (at least one religious ceremony/week), history of previous psychiatric care (outpatient, inpatient) and suicidal behavior (during the period of reference). ICD-10 clinical diagnosis, psychiatric history including outpatient care and previous inpatient stays prior to incarceration were recorded. We used most frequently speaking language instead of citizenship as an independent variable since this latter is contaminated by dual nationality issues (citizens born in immigrant families) and did not adequately reflect the cultural background of prison inmates. The collection of sociodemographic data was made by a board-certified psychiatrist (IDO), blind to the distinction between COT and regular inmates. All of the ICD-10 clinical diagnosis were made prospectively by two independent, board-certified psychiatrists (prior and during the hospital stay), blind to the scope of the study. Only cases with concordant psychiatric diagnoses were considered in this sample.

### Statistical analysis

Fisher exact, unpaired Student’s *t* and Mann–Whitney *U* tests were used to compare demographic and clinical variables according to the criminal status. Univariate and multiple logistic regression models were built to assess the determinants of COT versus usual detention (regular inmates). All statistical analysis were performed using Stata 16.1.

## Results

Group comparisons between COT and regular prison inmates are illustrated in Table 1. Several demographic parameters differentiate regular prison inmates from patients with COT. These latter were significantly older (*t* = − 2.10, DF: 107.60), less frequently married (Chi^2^ = 7.91, Fisher exact = 0.013), with better education attainment (Chi^2^ = 8.69, Fisher exact = 0.005). They were predominantly French-speaking (Chi^2^ = 6.12, Fisher exact = 0.016), and with regular religious practice (Chi^2^ = 5.04, Fisher exact = 0.035). As expected, history of psychiatric outpatient care (before conviction) was significantly more frequent in offenders with COT compared to regular prison inmates (Chi^2^ = 17.93, Fisher exact = 0.0001). Moreover, regular inmates had lower rates of annual admissions in UHPP compared to COT patients (Mann–Whitney *U* test, *z* = − 5.54, *p* = 0.0001). Group comparisons revealed two main differences in ICD-10 diagnoses. Unlike the significantly higher occurrence of adjustment disorders in regular prisoners (Chi^2^ = 17.84, Fisher exact = 0.0001), psychotic disorders were much more frequent in the group of offenders with COT (Chi^2^ = 13.99, Fisher exact = 0.0001). No group difference was found in the percentage of suicidal behavior, bipolar and depressive as well as personality disorders (Table 1).

**Table 1:** Demographic and clinical characteristics according to the criminal status. Statistical analysis was made using Fisher exact, unpaired Student’s *t* and Mann–Whitney *U* tests

	Criminal status			
	Usual detention	Court-ordered treatments	Total	*P*
*N*	139	61	200	
Age	31.7 ± 10.0	35.1 ± 10.7	32.8 ± 10.3	0.038
Sex women	19 (13.7%)	8 (13.1%)	27 (13.5%)	1.000
Marital status				0.013
Married	32 (23.0%)	4 (6.6%)	36 (18.0%)	
Separated–divorced–widowed	22 (15.8%)	13 (21.3%)	35 (17.5%)	
Single	85 (61.2%)	44 (72.1%)	129 (64.5%)	
Education (more than 9 years)	22 (15.8%)	21 (34.4%)	43 (21.5%)	0.005
Language French	85 (61.2%)	44 (72.1%)	129 (64.5%)	0.016
Language Arabic	43 (30.9%)	7 (11.5%)	50 (25.0%)	0.004
Language English	11 (7.9%)	10 (16.4%)	21 (10.5%)	0.083
Use of acute care				< 0.001
Single users	84 (60.4%)	16 (26.2%)	100 (50.0%)	
Frequent users	55 (39.6%)	45 (73.8%)	69 (34.5%)	
Religious group				0.048
None	46 (33.1%)	18 (29.5%)	64 (32.0%)	
Christian	37 (26.6%)	25 (41.0%)	62 (31.0%)	
Muslim	54 (38.8%)	15 (24.6%)	69 (34.5%)	
Other	2 (1.5%)	3 (4.9%)	5 (2.5%)	
Religious practice	6 (4.3%)	8 (13.1%)	14 (7.0%)	0.035
Psychiatric outpatient care	97 (69.8%)	59 (96.7%)	156 (78.0%)	< 0.001
Suicidal behavior	67 (48.2%)	24 (39.3%)	91 (45.5%)	0.282
Adjustment disorder	38 (27.3%)	1 (1.6%)	39 (19.5%)	< 0.001
Bipolar disorder	6 (4.3%)	3 (4.9%)	9 (4.5%)	1.000
Depressive disorder	11 (7.9%)	3 (4.9%)	14 (7.0%)	0.558
Personality disorder	40 (28.8%)	19 (31.1%)	59 (29.5%)	0.739
Psychotic disorder	41 (29.5%)	35 (57.4%)	76 (38.0%)	< 0.001

Among the variables included in group comparisons, older age, French language, higher education, and religious practice were all significantly associated with COT in univariate ordered logistic regression models. The negative association between marriage and COT was also confirmed. History of previous psychiatric outpatient care was strongly and positively related to COT. The occurrence of adjustment disorders was negatively related to COT whereas the inverse was true for psychotic disorders in univariate models. To take into account the interdependence of some independent variables, multivariable models were also considered. This analysis confirmed the above-mentioned associations between demographic factors and COT. However, when all diagnostic categories were included, the presence of personality and psychotic disorders were the only predictors of COT status (Table 2).

**Table 2:** Results of univariate (unadjusted OR) and multiple (adjusted OR) logistic regression associated with the criminal status (usual detention versus COT)

	Unadjusted		Adjusted	
Characteristics	Odds ratio	*P*	Odds ratio	*P*
Age	1.03 (1.00, 1.06)	0.035	1.05 (1.01, 1.11)	0.028
Language French	2.90 (1.21, 6.94)	0.017	4.07 (1.46, 11.32)	0.007
Language Arabic	0.29 (0.12, 0.69)	0.005	0.27 (0.10, 0.74)	0.011
Education more than 9 years	2.79 (1.39, 5.61)	0.004	1.57 (0.66, 3.73)	0.310
Marital status				
Married	0.24 (0.08, 0.73)	0.011	0.25 (0.07, 0.98)	0.047
Separated–divorced–widowed	1.14 (0.53, 2.48)	0.738	0.73 (0.24, 2.16)	0.566
Single	1.00 (1.00, 1.00)		1.00 (1.00, 1.00)	
Religious practice	3.35 (1.11, 10.11)	0.032	4.85 (1.00, 23.52)	0.050
Psychiatric outpatient care	12.77 (2.98, 54.72)	0.001	11.85 (2.36, 59.61)	0.003
Adjustment disorder	0.04 (0.01, 0.33)	0.002	0.17 (0.02, 1.82)	0.144
Personality disorder	1.12 (0.58, 2.15)	0.735	4.79 (1.13, 20.38)	0.034
Psychotic disorder	3.22 (1.72, 6.01)	0.000	5.19 (1.35, 19.94)	0.016

OR greater than 1 correspond to higher possibility to be under COT

## Discussion

Our data reveal major differences in sociodemographic and clinical patterns between COT patients and regular inmates, both admitted in the same acute psychiatric ward for crisis intervention. After controlling for the confounding effect of sociodemographic factors in multivariable models, they indicate that personality disorders and psychosis are independent determinants of COT. Given the relatively small sample size, we will discuss here only the differences present both in group comparisons and regression models.

Among demographic factors, patients with COT were older, with better education and knowledge of French as well as more frequent religious practice. One should keep in mind that in the Swiss law, COT are proposed by a psychiatric expert only when there is reasonable chance to reduce recidivism. As already indicated in previous studies, young ethnic-minority patients with low education are prone to negative assumptions about their care adherence and potential of change that may, in fact, preclude the proposal of COT [9, 18–22]. The presence of religious practice among COT patients may appear at first glance counterintuitive. However, considering himself religious is not only frequent among violent offenders [23], but is also associated with an increased risk of reconviction for non-sexual crimes [24]. It is thus likely that offenders with regular religious practice are overrepresented among COT since this measure mostly concerns serious crimes and multiple reconvictions. The association between single marital status and COT is in line with previous observations regarding the protective role of marriage against offending. One recent study reported that single status is a strong risk factor for criminal recidivism in community settings [25]. In the same line, the analysis of offenders with COT in Denmark during 1980–1992 revealed that they are predominantly single compared to regular prison inmates [26].

As one could expect, history of psychiatric outpatient care prior to conviction is much more frequent among offenders with COT. This reflects a long-standing vulnerability to mental disorders that has probably determined the proposal of inpatient treatment in a medium-security hospital. What is less expected is the marked difference in the use of acute psychiatric wards between the two populations. COT status is associated with a significant increase in the annual rate of admissions for crisis interventions. Previous observations of revolving door phenomenon were made in psychiatric hospitals. Drug addiction or discontinuation, crisis discharges, but also young age, low level of education, single status and recurrent suicidal thoughts have been all associated with heavy use of acute psychiatric wards [27–35]. Of note, none of these parameters could explain the group difference observed here since it persists in multivariate models controlling for age and marital status and education levels. Moreover, there are no significant differences in suicidal behavior between our groups and drug addiction or discontinuation were a priori excluded from our analysis. Two speculative explanations may explain this finding. Offenders with COT may be characterized by a long-lasting vulnerability to acute psychiatric episodes despite their intensive care program in medium-security hospitals. Alternatively, the COT status may be associated with increased use of compulsory admissions to acute psychiatric wards in order to discharge the teams of Curabilis that have to face severe and long-lasting behavioral disturbances. The relatively modest percentage of COT patients with suicidal behavior may be the consequence of the intensity of psychiatric care in Curabilis that is characterized by a continuous presence of mental health professionals in a daily basis. A previous study indicated that increasing conditions of deprivation and isolation are among the main predictors of suicide in prisoners [36].

From a clinical viewpoint, our findings show the frequent occurrence of adjustment disorders in regular prison inmates and predominance of psychotic disorders among COT patients. Both results are consistent with previous observations in the field [37–39]. In particular, the presence of adjustment disorders at the early phases of imprisonment is a well-known phenomenon with a high incidence mainly in solitary confinement [37]. Schizophrenia is one of the main determinants of COT in most Western countries [6, 15, 17, 40–42]. At first glance, the frequency of personality disorders was not significantly different between patients with COT and regular inmates. However, when controlling for the interaction between the different diagnoses, demographic factors and use of psychiatric services in multivariable models, a different picture emerges. Psychosis and personality disorders were independent determinants of COT. This finding is consistent with previous observations made in high and medium-security settings in England [42].

## Conclusions

Taken together, our results do not support the idea that COT introduce an artificial distinction among regular inmates with mental disorders. In conjunction with previous studies in psychiatric facilities [14–17], they imply that offenders with COT represent a clinically distinct group with an overrepresentation of personality and psychotic disorders compared to regular inmates with mental disorders. Moreover, they show that, at least in the Swiss penitentiary system, COT patients are less exposed to acculturation issues compared to regular inmates.

### Strengths and limitations

Strengths of the present study includes the presence of a single unit of acute psychiatric care in prison that decreases the variability in the criteria of admission, exclusion of cases with severe drug addiction and drug discontinuation, and use of multivariable models that make it possible to control for the interdependence between the clinical and demographic variables. Several limitations should, however, be considered. To be close to a real-life situation, clinical diagnosis was made using two independent clinicians blinded to the scope of the study but without use of standardized diagnostic questionnaires. Although the exclusion of cases with drug addiction and treatment discontinuation limits the generalizability of our findings, no previous studies showed significant differences in the occurrence of these conditions between COT and regular inmates. In the same line, only COT patients needing acute psychiatric care were considered leading to an overrepresentation of unstable cases that did not cover the full spectrum of COT. Binary data on religious practice and usual language may mask more complex realities in respect to the ethnic and cultural background of the inmates in both groups. Finally, and in order to save statistical power, no separate analysis by type of personality disorders was performed. Future studies in larger inmate samples including COT patients without acute care needs, standardized assessment of clinical diagnosis and demographic factors, and distinction between types of personality disorders are warranted to explore further the determinants of COT across the different legal and psychiatric care systems in Europe.

## Data Availability

The generated datasets are available by request to the corresponding author.

## Abbreviations

COT: Court-ordered treatments
UHPP: Unité hospitalière de psychiatrie pénitentiaire
